# Soret and Dufour effects on MHD squeezing flow of Jeffrey fluid in horizontal channel with thermal radiation

**DOI:** 10.1371/journal.pone.0266494

**Published:** 2022-05-19

**Authors:** Nur Azlina Mat Noor, Sharidan Shafie, Y. S. Hamed, Mohd Ariff Admon

**Affiliations:** 1 Department of Mathematical Sciences, Faculty of Science, Universiti Teknologi Malaysia, Johor Bahru, Johor, Malaysia; 2 Department of Mathematics and Statistics, College of Science, Taif University, Taif, Saudi Arabia; Central University of Karnataka, INDIA

## Abstract

The fluid flow with chemical reaction is one of well-known research areas in the field of computational fluid dynamic. It is potentially useful in the modelling of flow on a nuclear reactor. Motivated by the implementation of the flow in the industrial application, the aim of this study is to explore the time-dependent squeeze flow of magnetohydrodynamic Jeffrey fluid over permeable medium in the influences of Soret and Dufour, heat source/sink and chemical reaction. The presence of joule heating, joule dissipation and radiative heat transfer are analyzed. The flow is induced due to compress of two surfaces. Conversion of partial differential equations (PDEs) into ordinary differential equations (ODEs) is accomplished by imposing similarity variables. Then, the governing equations are resolved using Keller-box approach. The present outcomes are compared with previously outcomes in the literature to validate the precision of present outcomes. Both outcomes are shown in close agreement. The tabular and graphical results demonstrate that wall shear stress and velocity profile accelerate with the surfaces moving towards one another. Moreover, the concentration, temperature and velocity profiles decreasing for the increment of Hartmann numbers and Jeffrey fluid parameters. The impacts of heat generation/absorption, joule dissipation and Dufour numbers enhance the heat transfer rate and temperature profile. In contrast, the temperature profile drops and the heat transfer rate boosts when thermal radiation increases. The concentration profile decelerates, and the mass transfer rate elevates with raise in Soret number. Also, the mass transfer rate rises for destructive chemical reaction and contrary result is noted for convective chemical reaction.

## 1 Introduction

The movement of two parallel surfaces approaching one another is caused by external stress. The design of squeezing flow is adapted by researchers in the mechanical appliances such as modelling of oil flow in the bearings, lubrication system, injection moulding and hydraulic lift. The fundamental studies on the behaviour of flow in two surfaces was explored by Stefan [1]. Formulation model of squeeze flow with corresponded boundary conditions is derived via lubrication principle. Further, numerous works are done to analyse the behaviour of squeezing flow in the different geometries. The squeeze flow of elliptic and rectangle geometries was reported by Reynolds [2] and Archibald [3]. The previous works were formulated using Reynolds equation. However, Ishizawa [4] and Jackson [5] stated that Reynolds equation are not suitable in the analysis of squeeze flow in the high velocity and porous thrust bearings. Therefore, the fundamental mathematical model of squeeze flow has been revised and renewed in the various studies [6–11].

The research on boundary layer flow of non-Newtonian fluid attracted the interest of scientists due to the widespread in engineering application. Several models was proposed to discover the rheological behavior of non-Newtonian fluids. It is discovered that Jeffrey model is a simplest linear model with time derivatives as a substitute to convective derivatives [12]. It is categorized as shear thinning fluid because of high shear viscosity and yield stress [13]. The constitutive equation of Jeffrey fluid model was originally proposed by Pavlovskii [14] to investigate the dynamics of aqueous polymer solution. It is discovered that the addition of small amount of polymers in viscous fluid decrease the friction caused by drag force in the fluid flow [15]. Moreover, the model portrays the viscoelastic characteristics for the polymer industries by considering the relaxation and retardation parameter [16]. Common example of Jeffrey fluid is low-concentrated aqueous polymer solution [17]. Many authors used Jeffrey fluid model for studying the aqueous Polyacrylamide solution [18], blood flow in narrow arteries [19], movement of chyme in small intestine [20] and food bolus through esophagus [21] by applying the Jeffrey fluid model.

The studies of fluid flow over a porous medium has gain considerable attention due to the development of Darcy Law. A solid matrix with interconnected voids is known as permeable medium. It is explored in industrial and natural cases, for instances groundwater flow, petroleum reservoir rocks, engine coolant system and aircraft wings in the permeable cavities [22]. Moreover, the hydrodynamic of magnetic field in the electrical conducted fluid, magnetohydrodynamics (MHD) is widely reviewed because of its applications in MHD pump and generator. The hydromagnetic flow of Jeffrey fluid is discovered in many geometries. Hayat et al. [23] analysed the presence of injection or suction on MHD squeezing flow of Jeffrey fluid in the two permeable surfaces using homotopy analysis method (HAM) analytically. The squeeze flow of Jeffrey fluid with magnetic field on the stretching permeable lower plate with injection or suction was explored by Muhammad et al. [24]. Furthermore, Rao and Sreenadh [25] reviewed the magnetohydrodynamic flow of Jeffrey fluid on porous shrinking and stretching plate. The effect of hydromagnetic on Jeffrey fluid flow over porous medium in a circular tube was discovered by Nallapu and Radhakrishnamacharya [26]. Besides, the flow of Jeffrey fluid in the influence of magnetic field at a stagnation point was reviewed by Ahmad and Ishak [27]. The motion of the flow is due to stretched vertical plate. The problem was solved via Keller-box approach numerically.

The investigation on flow with viscous or joule dissipation is a subject of interest due to the several applications such as injection molding, high-rate extrusion and high temperature in polymer processes. The influence of joule dissipation is significant in the high velocity or viscosity fluid [28]. Hayat et al. [29] discussed the heat transfer of Jeffrey fluid on a stretched surface with joule dissipation. The convective heat transfer of Jeffrey fluid flow across a stretched plate with MHD, joule dissipation, joule heating, heat generation/absorption and radiative heat transfer was discovered by Ahmed et al. [30]. The impacts of joule dissipation and heating on the flow of Jeffrey fluid across a stretching surface was reviewed by Ahmad and Ishak [31]. Later, Zokri et al. [32] reported the numerical solution of Jeffrey fluid flow in a horizontal circular cylinder with the influence of viscous dissipation.

The electromagnetic waves emitted caused by the heat of substance is called thermal radiation. It is classified as one of the fundamental mechanisms of heat transfer. The sight of thermal radiation is common in the power generation, space vehicles, gas turbines and nuclear reactor chilling [33]. The effects of radiative heat transfer on Jeffrey fluid flow were reviewed for several geometries. Hayat et al. [34] explored the flow of Jeffrey fluid across a stretched sheet in the influence of thermal radiation at a stagnation point. The impacts of thermal radiation on squeezing flow of Jeffrey fluid in two disks was reported by Hayat et al. [35]. Furthermore, Hayat et al. [36] analysed the mixed convection flow of Jeffrey fluid past an inclined stretching sheet in the presence of thermal radiation. Kavita et al. [37] reported the oscillatory flow of MHD Jeffrey fluid on a vertical channel with radiative heat transfer.

The simultaneous thermal and mass transfer with chemical reaction has arises in many practical process including the flow in a desert and evaporation at the water surface. A chemical reaction among the fluid and the foreign particles takes place in the chemical industrial process. The common examples are the polymer production, the food processing and the manufacture of ceramics or glassware [38]. Alsaedi et al. [39] analysed the convective thermal transfer of Jeffrey fluid on a stretched surface with chemical reaction. The influences of radiative heat transfer and chemical reaction on oscillation flow of MHD Jeffrey fluid in horizontal channel was explored by Idowu et al. [40]. The flow and radiative heat transfer of Jeffrey fluid on a vertical porous surface with MHD and chemical reaction was discussed by Rao et al. [41]. Next, Saleem et al. [42] investigated the impacts of chemical reaction, heat source/sink and thermophoresis on convective thermal transfer of magneto-Jeffrey fluid on a rotated cone. The presence of chemical reaction on squeezing flow of Jeffrey nanofluid with magnetic field and velocity slip was examined by Noor et al. [43].

The phenomenon of thermal and mass transfer or double diffusion in a moving fluid plays a significant role in the field of petroleum reservoirs, nuclear waste disposal and air pollution [44]. It is noteworthy that the double diffusion process becomes more complicated due to the simultaneous occurrence of the driving potentials of heat and mass fluxes. The heat flux due to concentration gradient is indicated as diffusion-thermo or Dufour impact. Meanwhile, the mass flux due to temperature gradient is indicated as thermo-diffusion or Soret impact [45]. The Soret and Dufour term is found in non-dimensional concentration and energy equation, respectively. Generally, the Soret and Dufour impacts are not considered because the magnitude order is smaller than the impact specified by Fourier and Fick′s laws. Nevertheless, the impacts are considered when the presence of species at surface of fluid region have low density than the surrounding fluid [46]. Many researchers have analyzed the impacts of Soret and Dufour on flow with different geometries. Hayat et al. [47] explored the hydromagnetic flow of Casson fluid on a stretched surface with effect of Soret and Dufour. The mixed convection flow of nanofluid past a nonlinear stretching and shrinking surfaces in the influences of MHD, radiative heat transfer, Soret and Dufour was discussed by Pal et al. [48]. Further, Ullah et al. [49] examined the flow of Casson fluid across a nonlinear stretched plate with convective and slip boundaries. The presence of MHD, radiative heat transfer, joule dissipation, joule heating, heat generation or absorption, chemical reaction and Soret and Dufour was studied in the problem. The analysis of Soret and Dufour on squeeze flow of magneto-Casson fluid betweeen two surfaces with heat source/sink, joule heating and dissipation, chemical reaction and radiative heat transfer was investigated by Naduvinamani and Shankar [50].

The above cited papers reveal that the research focusing on squeeze flow of Jeffrey fluid over two surfaces are limited. Moreover, the thermal and mass transfer on squeezing flow of Jeffrey fluid is not yet covered. Thus, the aim of research is to explore Soret and Dufour impacts on time-dependent squeezing flow of MHD Jeffrey fluid embedded in a permeable medium with heat source/sink, joule heating and dissipation, chemical reaction and thermal radiation. The and later resolved through Keller-box technique. The numerical solutions are compared with published outputs in literature and shown in close agreement. Graphical outputs of concentration, temperature, and velocity profiles with correlated parameters are observed.

The fluid flow with chemical reaction is one of the most significant research areas in the field of computational fluid dynamic due to its industrial engineering applications. There are two categories of chemical reaction namely homogeneous and heterogeneous. The reaction is categorized as homogeneous or heterogeneous based on its occurrence in single phase (gaseous, liquid, or solid) or two phases (solid and gas, gas and liquid or solid and liquid), respectively [51]. Homogenous reaction occurs if the reactants and products are in the same phase while heterogeneous reactions have reactants in two or more phases [52].

The present study is mainly applied in the modelling of flow in a nuclear reactor. The presence of chemical reaction in the mathematical model is important to investigate the flow with nuclear reaction in the nuclear reactor. It is discovered that the lack of control of nuclear reaction may lead to the widespread contamination of air and water. Hence, the nuclear reaction flow is instantaneously stopped when the nuclear power plant accidents happen [53]. Moreover, the significance of MHD and permeable medium is analyzed in the fluid flow. Song et al. [54] discovered the capability of power conversion system raise in the presence of magnetohydrodynamics. The power conversion is essential to nuclear electric propulsion (NEP) system. NEP is simply electric propulsion in which the electricity is generated from a nuclear reactor. The safety standard enhance with the implementation of porous media concept as it accelerates the heat dissipation in the nuclear reactor [55]. The present study explores the following research questions:

1. How do the mathematical models for unsteady MHD squeezing flow in a porous medium with heat source/sink, joule heating and dissipation, chemical reaction and thermal radiation can be formulated?
2. How does the presence of Soret and Dufour will affect the temperature and concentration of Jeffrey fluid?
3. What is the variation of wall shear stress, heat and mass transfer with increasing in magnetic field, porosity, viscosity of Jeffrey fluid, heat source, thermal radiation, chemical reaction, Soret and Dufour impacts?

## 2 Mathematical formulation

**Fig 1:**
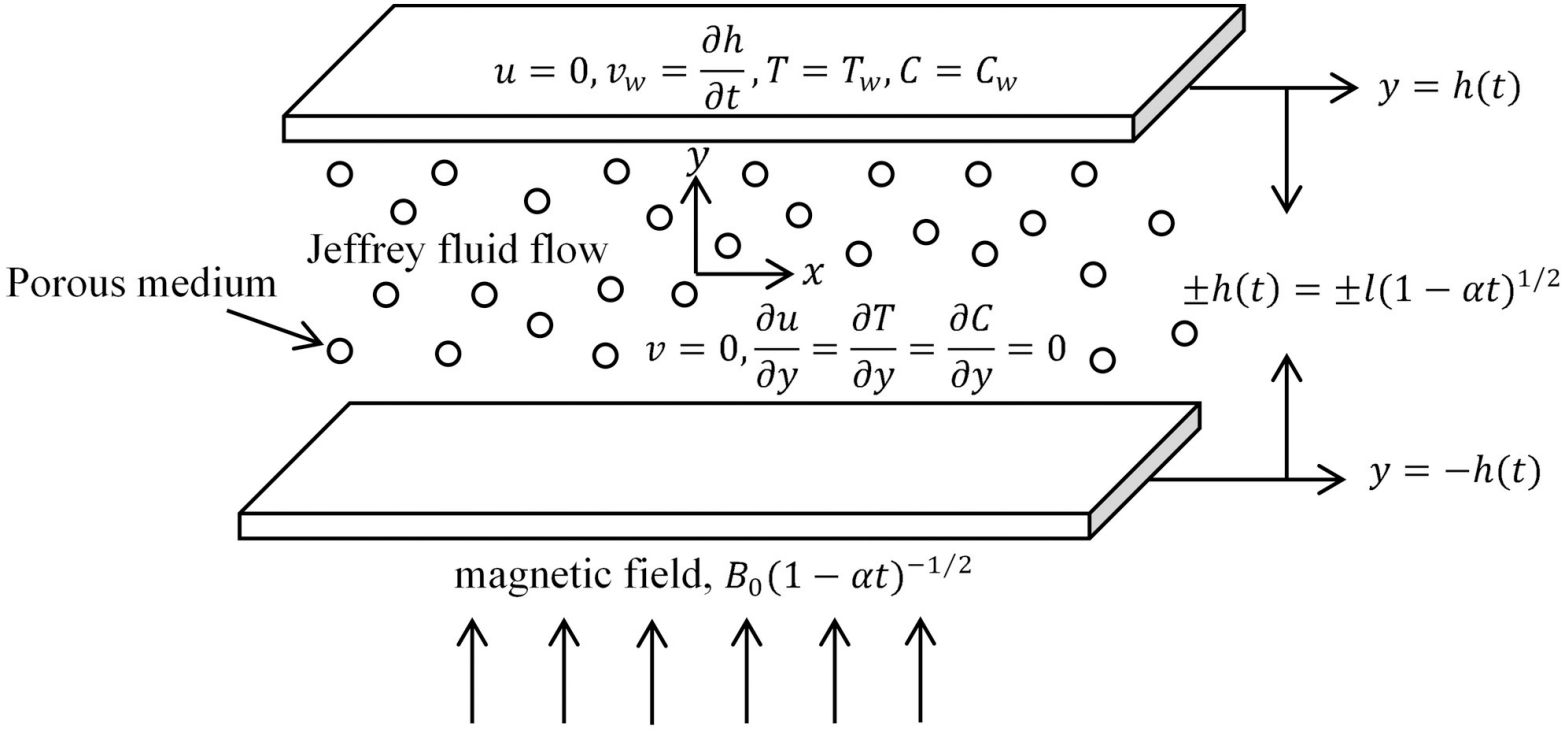
Schematic diagram of Jeffrey fluid between two squeezed plates embedded in a porous medium with transverse magnetic field.

The time dependent MHD flow of Jeffrey fluid induced by squeezing of two surfaces through porous medium with heat source/sink, Soret and Dufour and chemical reaction is studied. Also, the influences of joule dissipation, joule heating and thermal radiation are considered. The distance of two surfaces is *y* = ±*h*(*t*) = ±*l*(1 − *αt*)^1/2^. The two surfaces are moving further when *α* < 0 and the surfaces are moving closer when *α* > 0 till *t* = 1/*α* with velocity $v_{w}\left(t\right)=\frac{\partial h(t)}{\partial t}$. The lower plate is exerted with the magnetic field *B*(*t*) vertically [56]. Fig 1 depicts the geometrical model of Jeffrey fluid flow.

Referring to Jaluria [57], the constitutive relation of momentum equation for an incompressible fluid in two-dimensional form is given by

$$\begin{array}{c} \rho \frac{Du}{Dt}=divT+\rho F. \end{array}$$ (1)

Here, $\frac{D}{Dt}=\frac{\partial }{\partial t}+u\frac{\partial }{\partial x}+v\frac{\partial }{\partial y}$ represents the substantial derivative, *ρ* **F** denotes the body force and **T** is the Cauchy stress tensor. Based on Nadeem and Akbar [58], Cauchy stress tensor **T** of Jeffrey fluid is denoted by

$$\begin{array}{c} T=-pI+S, \end{array}$$ (2)

with **I** is identity tensor and *p* is pressure. The definition of extra stress tensor **S** is given by

$$\begin{array}{c} S=\frac{\mu }{1+\lambda_{1}}(A_{1}+\lambda_{2}\frac{DA_{1}}{Dt}), \end{array}$$ (3)

where **A**_1_ is the Rivilin-Ericksen tensor.

The derivation of energy equation is according to the first law of thermodynamics which stated energy is conserved. For any closed system, the energy remains constant and cannot be created nor destroyed over time. The energy equation in vector form is given by [59]

$$\begin{array}{c} \rho C_{p}\frac{DT}{Dt}=-\nabla \cdot q-\nabla \cdot j_{p.T}+\nabla \cdot q_{r}+\Phi +\frac{J^{2}}{\sigma }+Q(T-T_{\infty }), \end{array}$$ (4)

where **q** is the heat flux due to temperature gradient, **j**_*p*.*T*_ is the heat flux caused by thermophoretic effect, Φ is the viscous dissipation, $\frac{J^{2}}{\sigma }$ is Joule heating with the current density, **J**. The definition of **q** and **j**_*p*.*T*_ are expressed as

$$\begin{array}{c} q=-k_{f}\nabla T\;\text{and}\;j_{p.T}=-\frac{D_{m}k_{T}}{c_{s}}\rho \nabla C. \end{array}$$ (5)

Substitute Eq (5) into Eq (4) yields

$$\begin{array}{c} \rho C_{p}\frac{DT}{Dt}=\nabla \cdot (k_{f}\nabla T)+\nabla \cdot (\frac{D_{m}k_{T}}{c_{s}}\rho \nabla C)+\nabla \cdot q_{r}+\Phi +\frac{J^{2}}{\sigma }+Q(T-T_{\infty }). \end{array}$$ (6)

The radiative heat transfer, **q**_*r*_ based on Roseland approximation in two-dimensional form is denoted as [60]

$$\begin{array}{c} q_{r}=\frac{-4\sigma^{*}}{3k_{1}^{*}}[\frac{\partial T^{4}}{\partial x},\frac{\partial T^{4}}{\partial y}]. \end{array}$$ (7)

The small difference of temperature in the fluid is indicated as *T*^4^. The term *T*^4^ is expanded as linear function of temperature by Taylor’s series for *T*_∞_ and the higher order terms are ignored yields

$$\begin{array}{c} T^{4}≅4T_{\infty }^{3}T-3T_{\infty }^{4}. \end{array}$$ (8)

Then, substitue Eqs (7) and (8) into energy Eq (6) yields

$$\begin{array}{c} \rho C_{p}\frac{DT}{Dt}=k_{f}\nabla^{2}T+\frac{D_{m}k_{T}}{c_{s}}\rho \nabla^{2}C+\frac{16\sigma^{*}T_{\infty }^{3}}{3k_{1}^{*}}\nabla^{2}T+\Phi +\frac{J^{2}}{\sigma }+Q(T-T_{\infty }). \end{array}$$ (9)

The definition of **J** according to Ohm’s law is [28]

$$\begin{array}{c} J=\sigma [E+v\times B], \end{array}$$ (10)

with **B** is the total magnetic field and **E** is the electric field. It is assumed that **E** is ignored because there are no polarization and external applied electric field. Then, Eq (8) becomes

$$\begin{array}{c} \begin{array}{cc} J & =\sigma [v\times B]\\ & =<-\sigma B^{2}u,-\sigma B^{2}v,0>. \end{array} \end{array}$$ (11)

Substitute Eq (11) into the energy Eq (9) yields

$$\begin{array}{c} \begin{array}{cc} \rho C_{p}\frac{DT}{Dt} & =k_{f}\nabla^{2}T+\frac{D_{m}k_{T}}{c_{s}}\rho \nabla^{2}C+\frac{16\sigma^{*}T_{\infty }^{3}}{3k_{1}^{*}}\nabla^{2}T+\Phi -\sigma B^{2}u^{2}-\sigma B^{2}v^{2}\\ & +Q(T-T_{\infty }). \end{array} \end{array}$$ (12)

The viscous dissipation term Φ is expressed as [61]

$$\begin{array}{c} \Phi =\mu (1+\frac{1}{\lambda_{1}})[4{\left(\frac{\partial u}{\partial x}\right)}^{2}+{\left(\frac{\partial u}{\partial y}+\frac{\partial v}{\partial x}\right)}^{2}]. \end{array}$$ (13)

Therefore, the energy Eq (12) can be written as follows

$$\begin{array}{c} \begin{array}{cc} \rho C_{p}\frac{DT}{Dt} & =k_{f}\nabla^{2}T+\frac{16\sigma^{*}T_{\infty }^{3}}{3k_{1}^{*}}\nabla^{2}T+\mu (1+\frac{1}{\lambda_{1}})[4{\left(\frac{\partial u}{\partial x}\right)}^{2}+{\left(\frac{\partial u}{\partial y}+\frac{\partial v}{\partial x}\right)}^{2}]\\ & -\sigma B^{2}u^{2}-\sigma B^{2}v^{2}+Q(T-T_{\infty })+\frac{D_{m}k_{T}}{c_{s}}\rho \nabla^{2}C, \end{array} \end{array}$$ (14)

or

$$\begin{array}{c} \begin{array}{cc} \frac{DT}{Dt} & =\alpha_{f}\nabla^{2}T+\alpha_{f}\frac{16\sigma^{*}T_{\infty }^{3}}{3k_{1}^{*}}\nabla^{2}T+\frac{\nu_{f}}{C_{p}}(1+\frac{1}{\lambda_{1}})[4{\left(\frac{\partial u}{\partial x}\right)}^{2}+{\left(\frac{\partial u}{\partial y}+\frac{\partial v}{\partial x}\right)}^{2}]\\ & -\frac{\sigma B^{2}u^{2}}{\rho C_{p}}-\frac{\sigma B^{2}v^{2}}{\rho C_{p}}+\frac{Q(T-T_{\infty })}{\rho C_{p}}+\frac{D_{m}k_{T}}{c_{s}C_{p}}\nabla^{2}C. \end{array} \end{array}$$ (15)

The governing equations of Jeffrey fluid are reduced to the following equations using boundary layer approximation

$$\begin{array}{c} \frac{\partial u}{\partial x}+\frac{\partial v}{\partial y}=0, \end{array}$$ (16)

$$\begin{array}{c} \begin{array}{cc} \frac{\partial u}{\partial t}+u\frac{\partial u}{\partial x}+v\frac{\partial u}{\partial y}= & \nu_{f}(1+\frac{1}{\lambda_{1}})\frac{\partial^{2}u}{\partial y^{2}}+\nu_{f}\frac{\lambda_{2}}{1+\lambda_{1}}(\begin{array}{c} \frac{\partial^{3}u}{\partial t\partial y^{2}}+u\frac{\partial^{3}u}{\partial x\partial y^{2}}\\ +v\frac{\partial^{3}u}{\partial y^{3}}-\frac{\partial u}{\partial x}\frac{\partial^{2}u}{\partial y^{2}}+\frac{\partial u}{\partial y}\frac{\partial^{2}u}{\partial x\partial y} \end{array})\\ & -\frac{\sigma B^{2}\left(t\right)}{\rho_{f}}u-\nu_{f}(1+\frac{1}{\lambda_{1}})\frac{\varphi }{k_{1}\left(t\right)}, \end{array} \end{array}$$ (17)

$$\begin{array}{c} \begin{array}{cc} \frac{\partial T}{\partial t}+u\frac{\partial T}{\partial x}+v\frac{\partial T}{\partial y}= & \alpha_{f}(1+\frac{16\sigma^{*}T_{\infty }^{3}}{3k_{f}k_{1}^{*}})\frac{\partial^{2}T}{\partial y^{2}}+\frac{\nu_{f}}{c_{f}}(1+\frac{1}{\lambda_{1}})[4{\left(\frac{\partial u}{\partial x}\right)}^{2}+{\left(\frac{\partial u}{\partial y}\right)}^{2}]\\ & +\frac{\sigma B^{2}\left(t\right)}{{\left(\rho c\right)}_{f}}u^{2}+\frac{Q(t)}{{\left(\rho c\right)}_{f}}T+\frac{D_{m}k_{T}}{c_{s}c_{p}}\frac{\partial^{2}C}{\partial y^{2}}. \end{array} \end{array}$$ (18)

$$\begin{array}{c} \begin{array}{c} \frac{\partial C}{\partial t}+u\frac{\partial C}{\partial x}+v\frac{\partial C}{\partial y}=D_{m}\frac{\partial^{2}C}{\partial y^{2}}+\frac{D_{m}k_{T}}{T_{m}}\frac{\partial^{2}T}{\partial y^{2}}-k_{c}\left(t\right)C. \end{array} \end{array}$$ (19)

The corresponded boundary conditions (BCs) are

$$\begin{array}{c} u=0,v=v_{w}=\frac{\partial h(t)}{\partial t},T=T_{w},C=C_{w},\;\;\;\;\;\;\;\text{at}\;y=h\left(t\right), \end{array}$$ (20)

$$\begin{array}{c} \frac{\partial u}{\partial y}=0,\frac{\partial^{3}u}{\partial y^{3}}=0,v=0,\frac{\partial T}{\partial y}=0,\frac{\partial C}{\partial y}=0,\;\;\;\;\;\;\;\;\text{at}\;\;y=0. \end{array}$$ (21)

The non-dimensional variables are applied to simplify PDEs into ODEs [62];

$$\begin{array}{c} \begin{array}{cc} & u=\frac{\alpha x}{2(1-\alpha t)}f^{\prime }\left(\eta \right),v=\frac{\alpha l}{2\sqrt{\left(1-\alpha t\right)}}f\left(\eta \right),\eta =\frac{y}{l\sqrt{\left(1-\alpha t\right)}},\\ & \theta =\frac{T}{T_{w}},\phi =\frac{C}{C_{w}}. \end{array} \end{array}$$ (22)

Substituting dimensionless variables (22) into Eqs (17), (18) and (19) gives the subsequent forms

$$\begin{array}{c} \begin{array}{cc} (1+\frac{1}{\lambda_{1}})f^{iv}- & S(\eta f^{\prime \prime \prime }+3f^{\prime \prime }+f^{\prime }f^{\prime \prime }-ff^{\prime \prime \prime })\\ & +(1+\frac{1}{\lambda_{1}})\frac{De}{2}\left(\eta f^{v}+5f^{iv}+2f^{\prime \prime }f^{\prime \prime \prime }-f^{\prime }f^{iv}-ff^{v}\right)-Ha^{2}f^{\prime \prime }\\ & -(1+\frac{1}{\lambda_{1}})\frac{1}{Da}f^{\prime \prime }=0, \end{array} \end{array}$$ (23)

$$\begin{array}{c} \begin{array}{cc} \frac{1}{Pr}(1+\frac{4}{3}R_{d}) & \theta^{\prime \prime }+S\left(f\theta^{\prime }-\eta \theta^{\prime }+\gamma \theta \right)\\ & +Ec[(1+\frac{1}{\lambda_{1}})\left[{\left(f^{\prime \prime }\right)}^{2}+4\delta^{2}{\left(f^{\prime }\right)}^{2}+Ha^{2}{\left(f^{\prime }\right)}^{2}\right]]+Du\phi^{\prime \prime }=0, \end{array} \end{array}$$ (24)

$$\begin{array}{c} \frac{1}{Sc}\phi^{\prime \prime }+S\left(f\phi^{\prime }-\eta \phi^{\prime }\right)+Sr\theta^{\prime \prime }-R\phi =0,\;\;\;\;\;\;\;\;\;\;\;\;\;\;\;\; \end{array}$$ (25)

with dimensionless BCs

$$\begin{array}{c} f\left(\eta \right)=0,f^{\prime \prime }\left(\eta \right)=0,f^{iv}\left(\eta \right)=0,\theta^{\prime }\left(\eta \right)=0,\phi^{\prime }\left(\eta \right)=0,\;\text{at}\;\eta =0, \end{array}$$ (26)

$$\begin{array}{c} f\left(\eta \right)=1,f^{\prime }\left(\eta \right)=0,\theta \left(\eta \right)=1,\phi \left(\eta \right)=1,\;\;\;\;\;\;\;\text{at}\;\eta =1. \end{array}$$ (27)

The pertinent terms in the dimensionless equations are described by

$$\begin{array}{c} \begin{array}{cc} & S=\frac{\alpha l^{2}}{2\nu_{f}},Ha=lB_{0}\sqrt{\frac{\sigma }{\rho_{f}\nu_{f}}},Da=\frac{k_{0}}{\varphi l^{2}},De=\frac{\alpha \lambda_{2}}{1-\alpha t},\delta =\frac{l}{x}{\left(1-\alpha t\right)}^{1/2},\\ & Pr=\frac{\nu_{f}}{\alpha_{f}},R_{d}=\frac{4\sigma^{*}T_{\infty }^{3}}{k_{f}k_{1}^{*}},Ec=\frac{\alpha^{2}x^{2}}{4c_{f}T_{w}{\left(1-\alpha t\right)}^{2}},\gamma =\frac{2Q_{0}}{\alpha {\left(\rho c\right)}_{f}},\\ & Du=\frac{D_{m}k_{T}C_{w}}{c_{s}c_{p}\nu_{f}T_{w}},Sr=\frac{D_{m}k_{T}T_{w}}{T_{m}\nu_{f}C_{w}},Sc=\frac{\nu_{f}}{D_{m}},R=\frac{k_{2}l^{2}}{\nu_{f}}. \end{array} \end{array}$$ (28)

Physically, the motion of two surfaces is indicated by squeeze term, with *S* > 0 portrays the surfaces approaching nearer and *S* < 0 portrays the surfaces separating apart. Moreover, Darcy, Hartmann and Deborah terms are implemented to manage the velocity field. The temperature is regulated by radiative heat transfer, Eckert and heat generation or absorption terms. Further, the impacts of Dufour and Soret are explored in concentration and temperature graphs. The addition of chemical reaction term is examined in concentration profile.

## 3 Results and discussion

The governing Eqs (23) to (25) in corresponded BCs (26) and (27) are resolved using Keller-box procedure numerically. The four steps to get the numerical results are

1. The conversion of ODEs to a system of 1^*st*^ order equations.
2. The discretization of 1^*st*^ order equations into the form of finite difference via central difference method.
3. The linearization of nonlinear equations with Newton’s method and addressed in the form of matrix-vector.
4. The linear system is solved using block tri-diagonal elimination technique.

The algorithm based on Keller-box approach is developed in MATLAB for the iterative computation to obtain numerical and graphical outputs. The proper values of the step size Δ*η* = 0.01 and thickness of boundary layer *η*_∞_ = 1 are compulsory to get the accurate outputs. The convergence criterion is referred to the variation in the current and former outputs of concentration, temperature and velocity. Calculation is ended when the numerical outputs converging to 10^−5^ [63].

The numerical calculation is done to examine the impacts of *S*, *Ha*, λ_1_, *De*, *Da*, *Pr*, *Ec*, *γ*, *R*_*d*_, *R*, *Sc*, *Du* and *Sr* on concentration, temperature and velocity profiles. The algorithm built in the MATLAB is validated by comparison with the present results of −*f*′′(1), −*θ*′(1) and −*ϕ*′(1) with previous existing results by Naduvinamani and Shankar [50] as limiting cases. Both outputs are shown in the good agreement as displayed in Table 1.

**Table 1:** Numerical values of −*f*′′(1), −*θ*′(1) and −*ϕ*′(1) for S when λ_1_ → ∞, *Da* → ∞, *De* = 10^−10^, *R*_*d*_ = *Ha* = *γ* = *Du* = *Sr* = 0, *δ* = 0.1 and *R* = *Sc* = *Pr* = *Ec* = 1.

*S*	Naduvinamani and Shankar [50]			Present results		
	−*f*′′(1)	−*θ*′(1)	−*ϕ*′(1)	−*f*′′(1)	−*θ*′(1)	−*ϕ*′(1)
2.0	4.167389	3.118551	0.701813	4.167412	3.118564	0.701819
0.5	3.336449	3.026324	0.744224	3.336504	3.026389	0.744229
0.01	3.007134	3.047092	0.761225	3.007208	3.047166	0.761229
-0.5	2.617404	3.129491	0.781402	2.617512	3.129556	0.781404
-1.0	2.170091	3.319899	0.804559	2.170255	3.319904	0.804558

Limiting cases analysis is conducted by setting the quantity of interest to a specific extreme value (usually 0 or ∞). For instance, the present work is reduced to the pioneer work by Naduvinamani and Shankar [50] as shown in Table 1. The values of dimensionless parameter is setting as λ_1_ → ∞, *Da* → ∞, *R*_*d*_ = *Ha* = *γ* = *Du* = *Sr* = 0, *De* = 10^−10^, *δ* = 0.1 and *Sc* = *Ec* = *R* = *Pr* = 1 when input in the MATLAB program. All the results are discovered in excellent agreement. Hence, it is proven that the Keller-box scheme used to get the present outcomes is accurate and acceptable.

**Fig 2:**
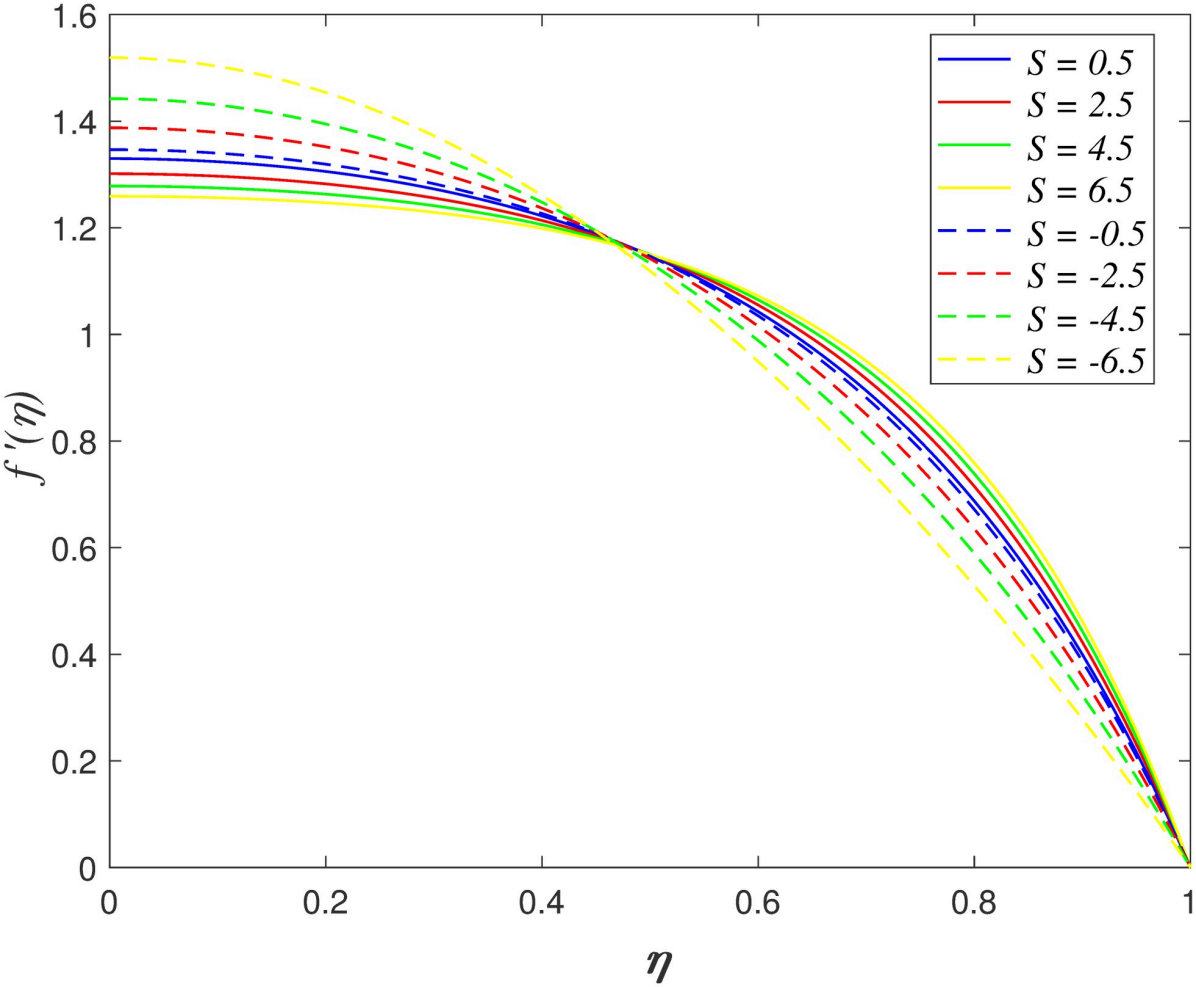
Impact of *S* on *f*′(*η*).

**Fig 3:**
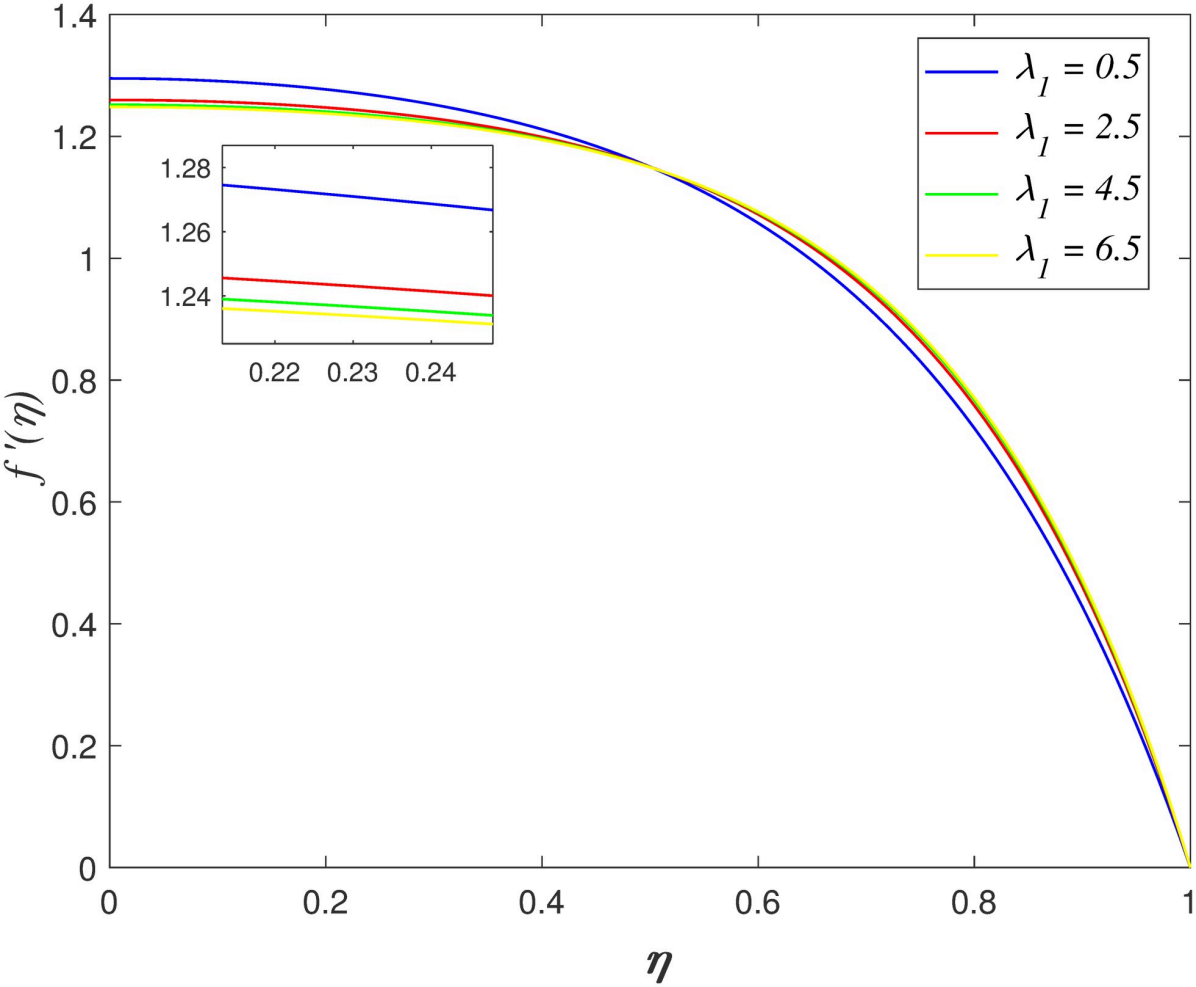
Impact of λ_1_ on *f*′(*η*).

**Fig 4:**
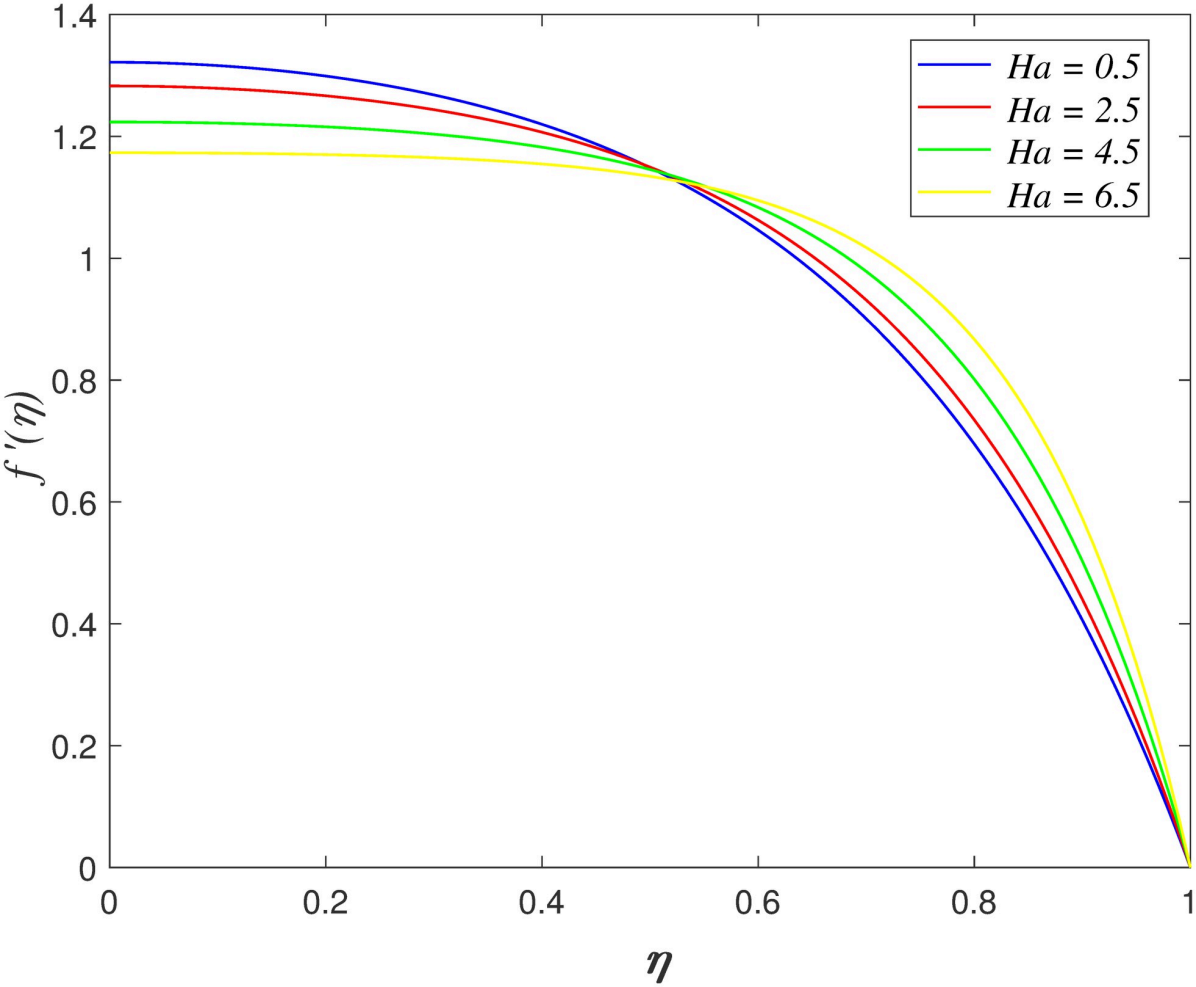
Impact of *Ha* on *f*′(*η*).

**Fig 5:**
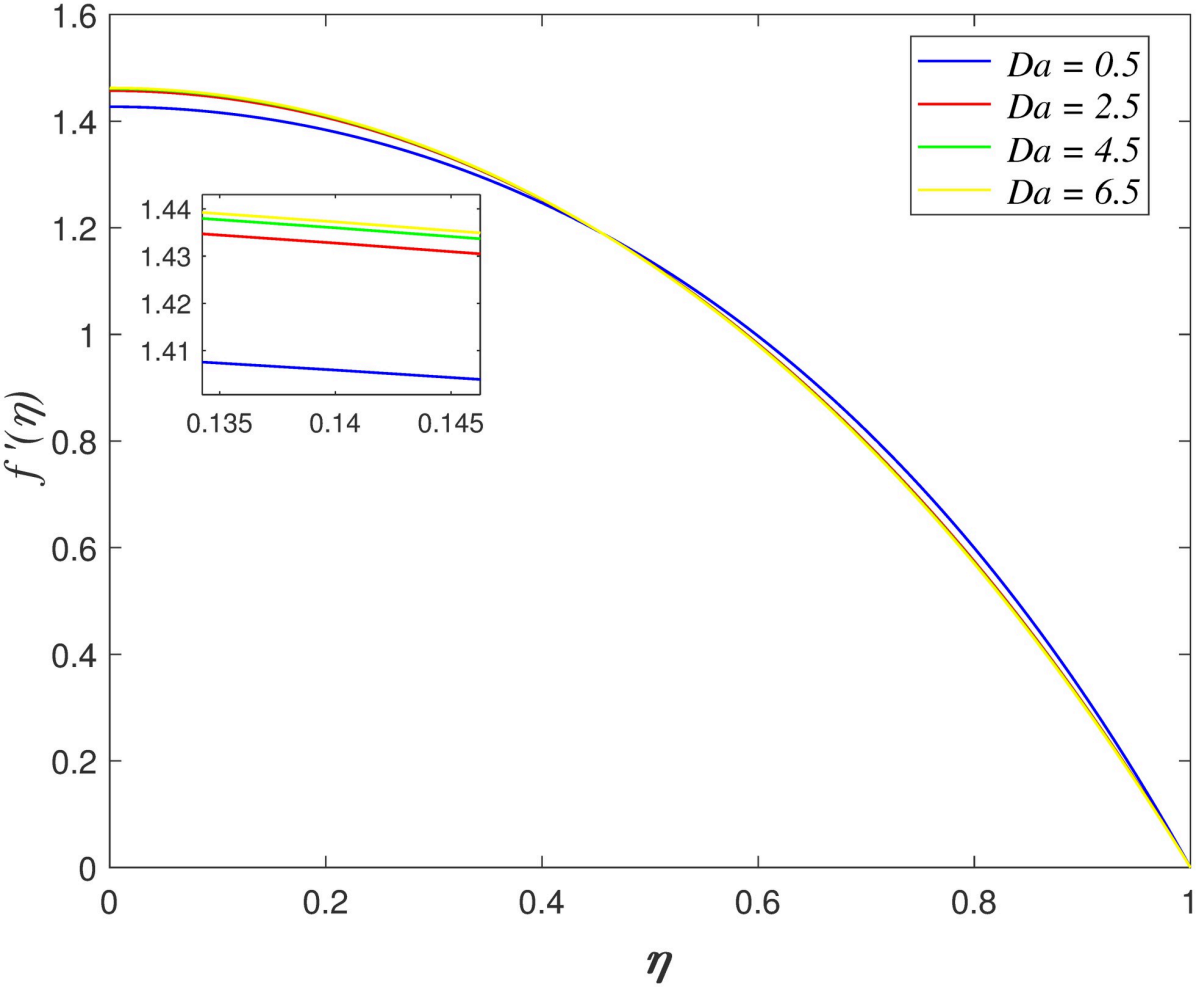
Impact of *Da* on *f*′(*η*).

**Fig 6:**
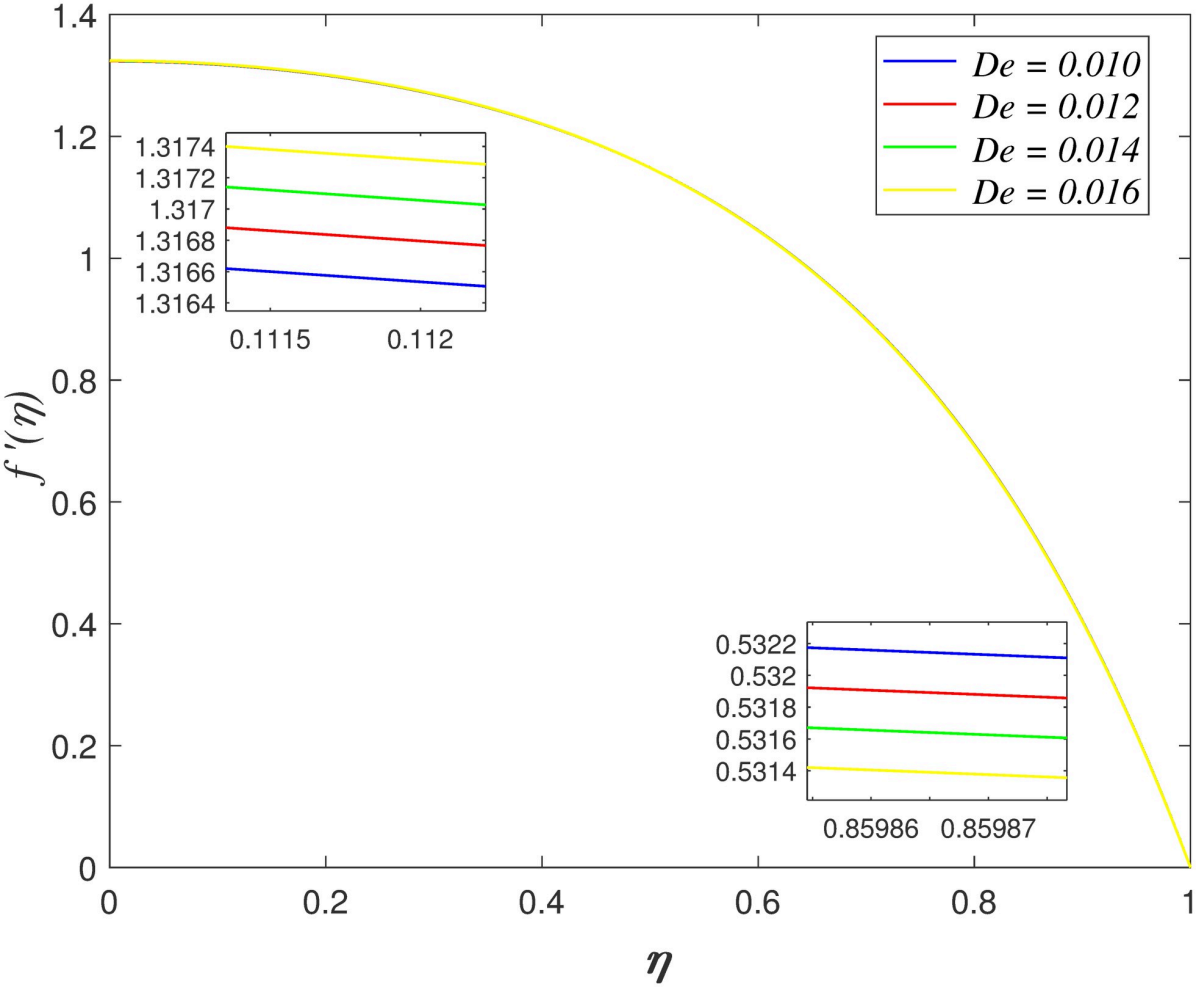
Impact of *De* on *f*′(*η*).

The impacts of *S*, λ_1_, *Ha*, *Da* and *De* on axial velocity is plotted in Figs 2 to 6. The motion of surfaces closer is described by *S* > 0 and the movement of surfaces further is described by *S* < 0. Fig 2 shows the effects of *S* on axial velocity. It is noticed that boundary range close on the below surface is 0 ≤ *η* < 0.5 and the boundary range near on the above surface is 0.5 ≤ *η* ≤ 1. It is discovered that the velocity decreasing as *η* < 0.5 and it elevates as *η* ≥ 0.5 with *S* > 0. In contrary, the velocity enhancing as *η* < 0.5 and it decelerates as *η* ≥ 0.5 with *S* < 0. The flow cross the confined channel accelerates as the surfaces approaching closer, which resulting in the velocity profile accelerates. In contrast, the deceleration of velocity is due to the flow confronts higher resistance in the wider channel. There is cross flow behaviour at the midpoint of channel. The squeezing parameter does not affect the fluid velocity at *η*_*c*_ = 0.5 (critical point). The similar behaviour of velocity profile with the variation of *S* is shown in the work by Naduvinamani and Shankar [50]. The influence of λ_1_ on axial velocity is displayed in Fig 3. The velocity slowing down as *η* ≤ 0.45 and it accelerates as *η* > 0.45 as λ_1_ increasing. It is noticed that the flow decelerates due to the high fluid viscosity and the intermolecular forces of fluid molecules enhance as λ_1_ increases. Fig 4 portrays the effect of *Ha* on axial velocity. The velocity reduces as *η* ≤ 0.45 and it enhances as *η* > 0.45 with *Ha* rises. Lorentz force is generated by imposing the magnetic field on the electrical conducted fluid. The opposition on the flow elevates with the presence of Lorentz force and consequently, decelerate the velocity in the boundary area. The behaviour of velocity profiles when varying λ_1_ and *Ha* parameters is the same with the results of Hayat et al. [23]. The impact of *Da* on axial velocity is explored in Fig 5. The velocity increases as *η* ≤ 0.45 and it declining as *η* > 0.45 with enhance in *Da*. The increment of Darcy parameter boosts the permeability of medium and thus, accelerates the flow through permeable medium at the centre of boundary area. Fig 6 shows the influences of *De* on axial velocity. The velocity enhances at the below surface and it decreasing at the above surface with rising in *De*. The ratio of retardation time and the observation time is denoted by Deborah term. Retardation time is the delayed reaction to the internal stress or delay of elasticity. The fluid has high viscosity because the retardation time is longer as *De* increases. It implies that the enhancement of intermolecular forces of fluid molecules, resulting in the flow nearer the upper plate slows down. The variation of *De* on velocity profile is the same as illustrated in Muhammad et al. [24].

**Fig 7:**
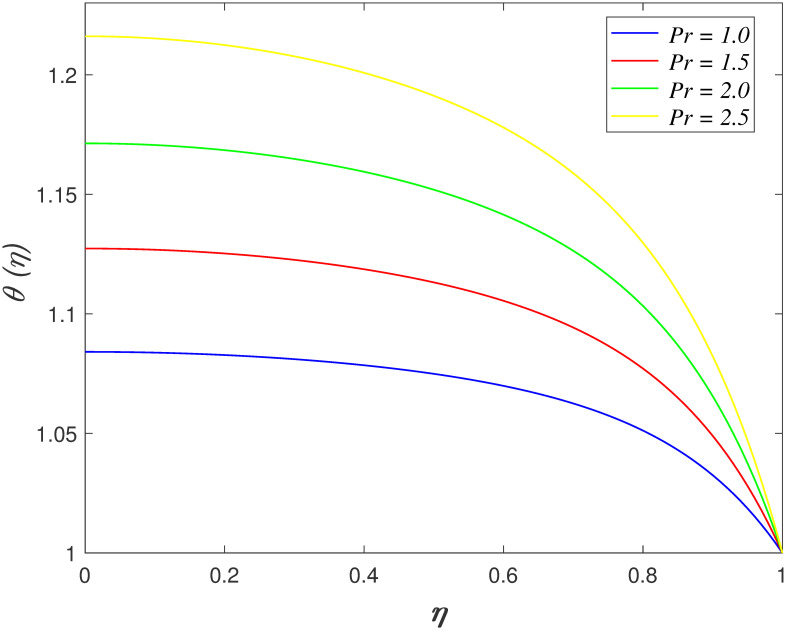
Impact of *Pr* on *θ*(*η*).

**Fig 8:**
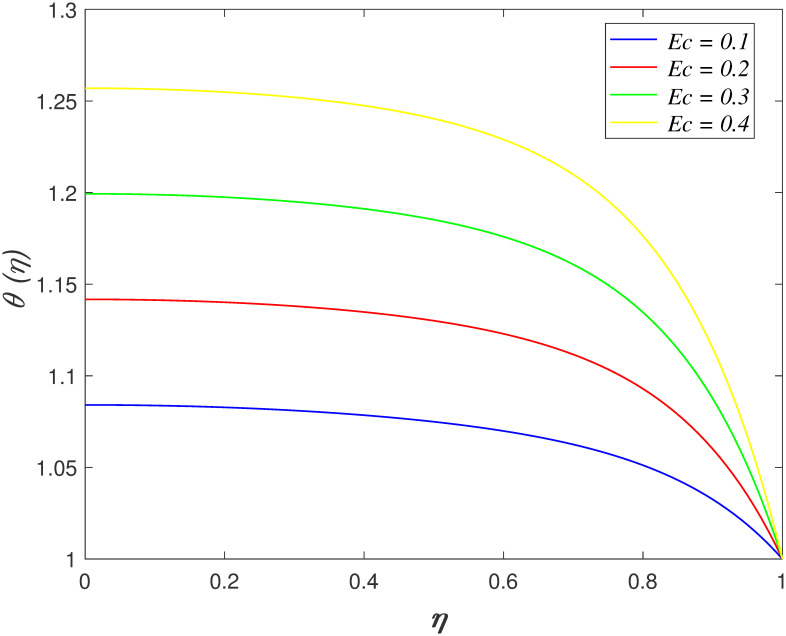
Impact of *Ec* on *θ*(*η*).

**Fig 9:**
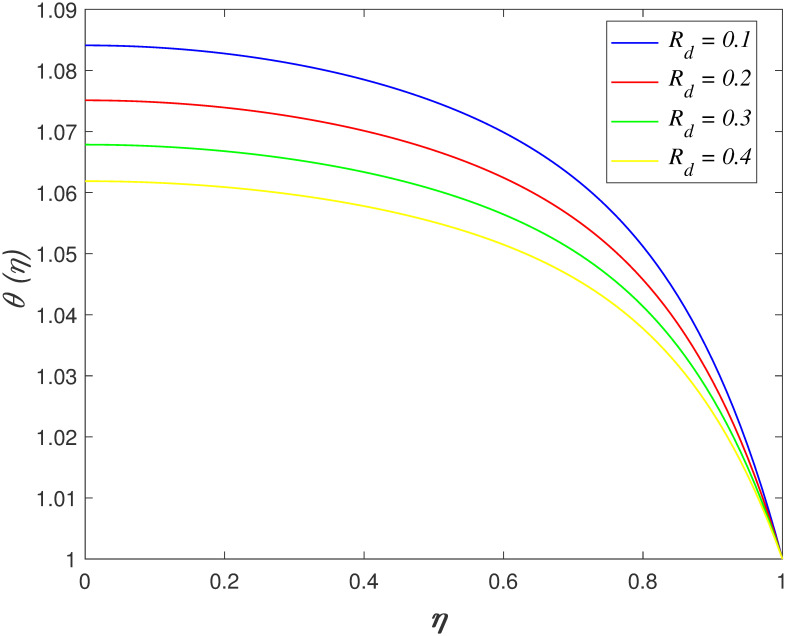
Impact of *R*_*d*_ on *θ*(*η*).

**Fig 10:**
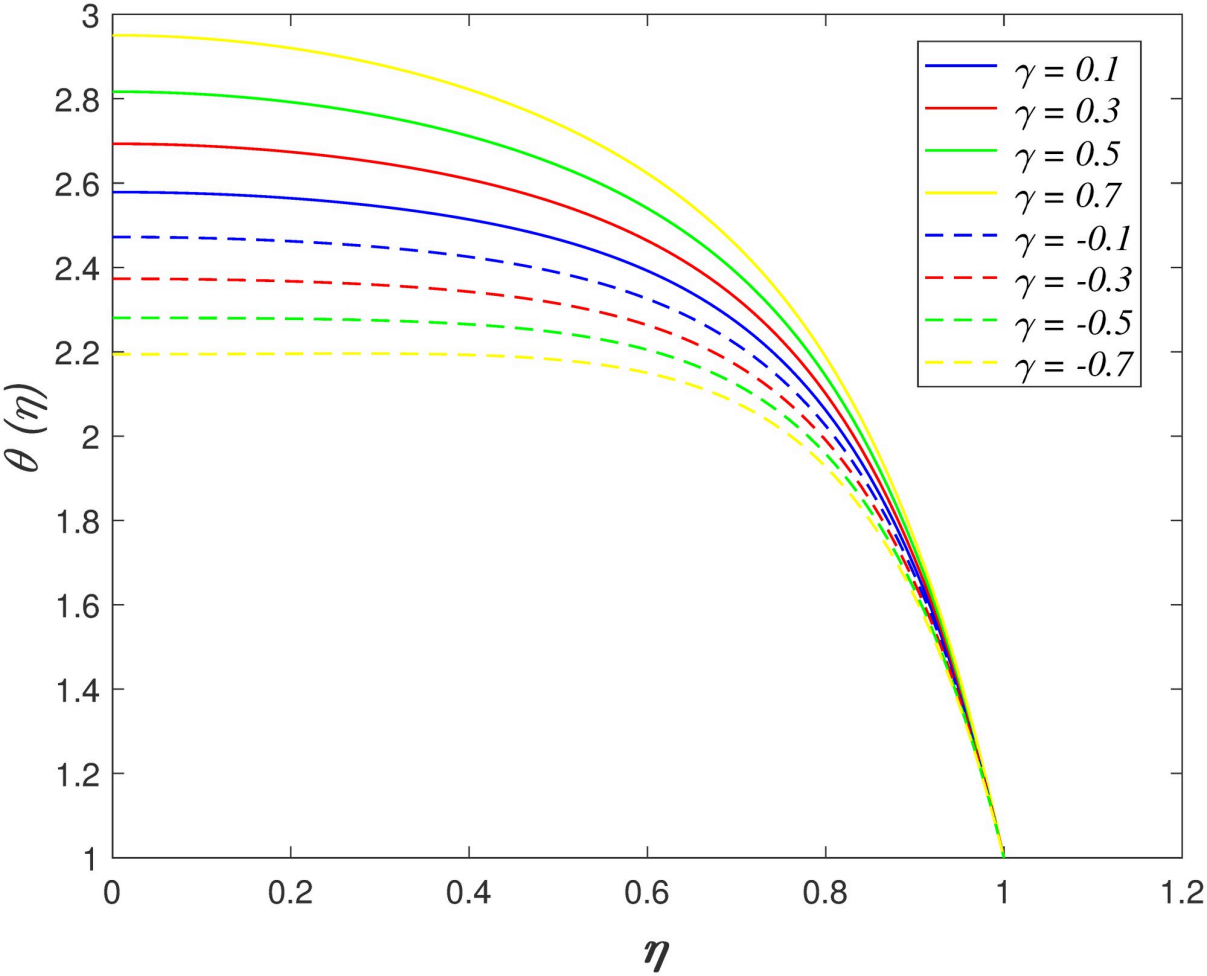
Impact of *γ* on *θ*(*η*).

**Fig 11:**
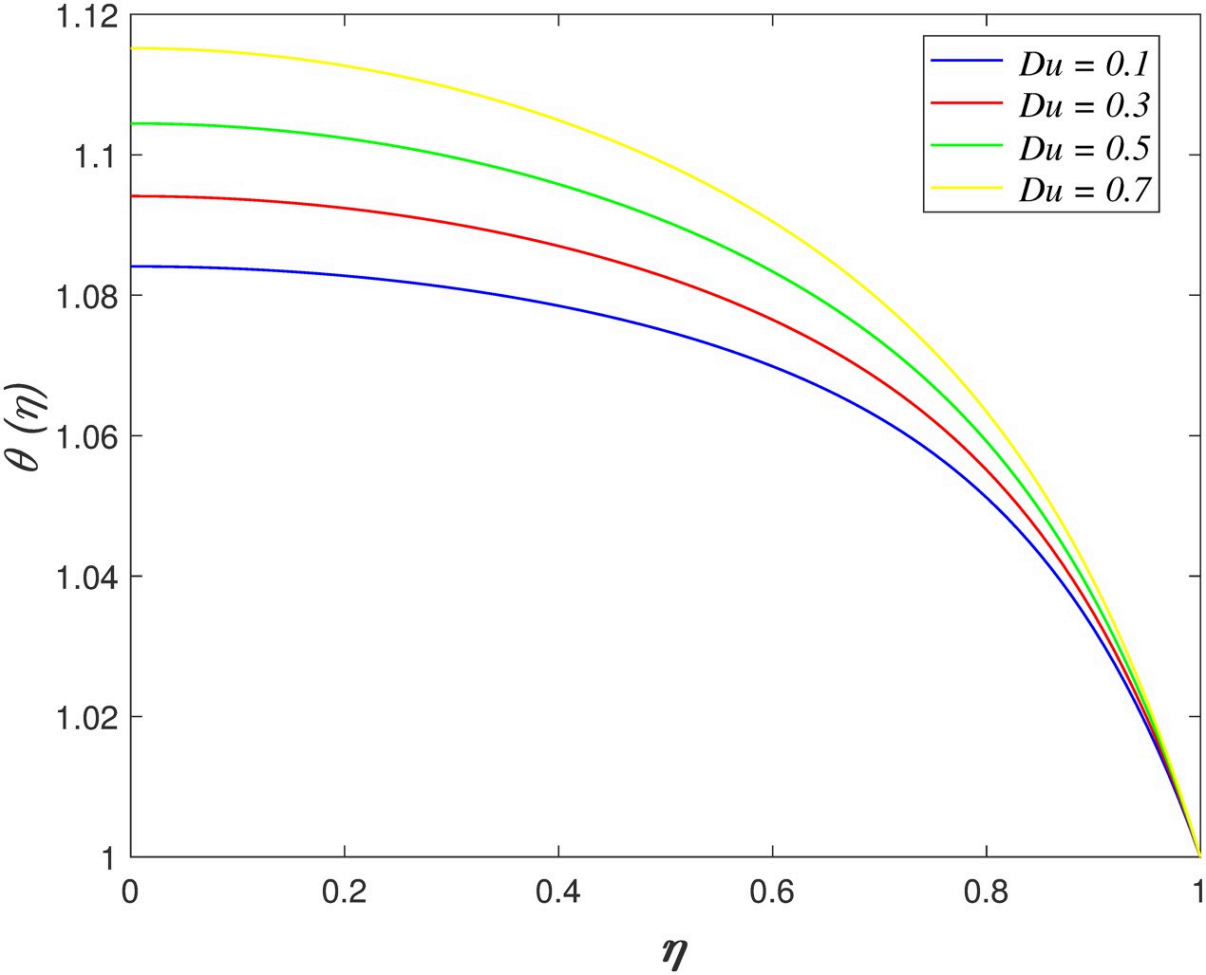
Impact of *Du* on *θ*(*η*).

**Fig 12:**
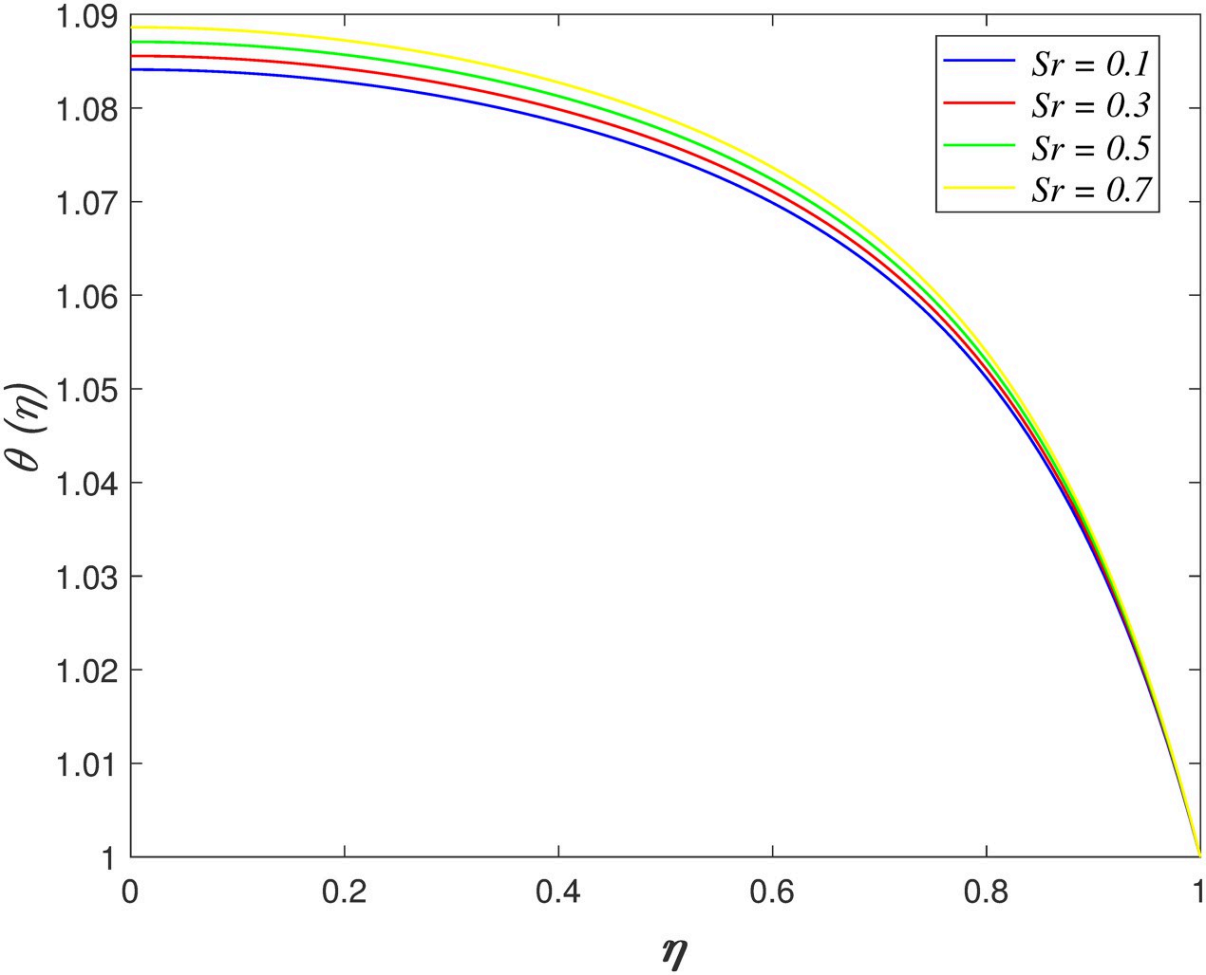
Impact of *Sr* on *θ*(*η*).

Fig 7 portrays the impacts of *Pr* on temperature region. The flow temperature elevating when *Pr* rises. The raise in *Pr* indicates the larger specific heat capacity of fluid. The temperature increases because of the heat absorption elevates in the fluid flow. The variation of *Ec* on temperature region is presented in Fig 8. It is discovered that temperature field boosts with the increment of *Ec*. The viscous dissipation is represented by *Ec*. The heat generated by the internal friction of fluid particles rises as *Ec* increases. Hence, it has resulting the increment of the temperature field. Fig 9 illustrates the influences of *R*_*d*_ on temperature region. The transfer of thermal energy caused by the emission of electromagnetic waves from heated substance is described as thermal radiation. The reduction of temperature profile occurs because the heat transfer from flow area to the surfaces elevates for increasing *R*_*d*_ values. The effect of *γ* on temperature region is indicated in Fig 10. The heat absorption and heat generation case are characterized by *γ* < 0 and *γ* > 0, respectively. The temperature profile drops when *γ* < 0 and it rising when *γ* > 0. The heat generation increase the thermal energy of fluid, which cause the temperature profile increases. Meanwhile, a contrary behaviour is discovered in the heat absorption case. Fig 11 discovers the impact of *Du* on temperature region. The temperature profile enhances with raise in *Du*. This behavior is due to the reason that the kinematic viscosity in the vicinity of boundary flow decreases. The resistance to the fluid flow is measured by kinematic viscosity. The kinetic energy of fluid particles accelerates because the resistance in the flow slowing down. The influences of *Sr* on temperature region is depicted in Fig 12. It is discovered that the increment of *Sr* boost the temperature profile. Soret number is inversely proportional to the kinematic viscosity. It implies that the fluid confronts less resistance and the kinetic energy in the boundary region increases. The variation of temperature profiles for *Pr*, *Ec*, *R*_*d*_, *γ*, *Du* and *Sr* is similar as shown in Naduvinamani and Shankar [50] work.

**Fig 13:**
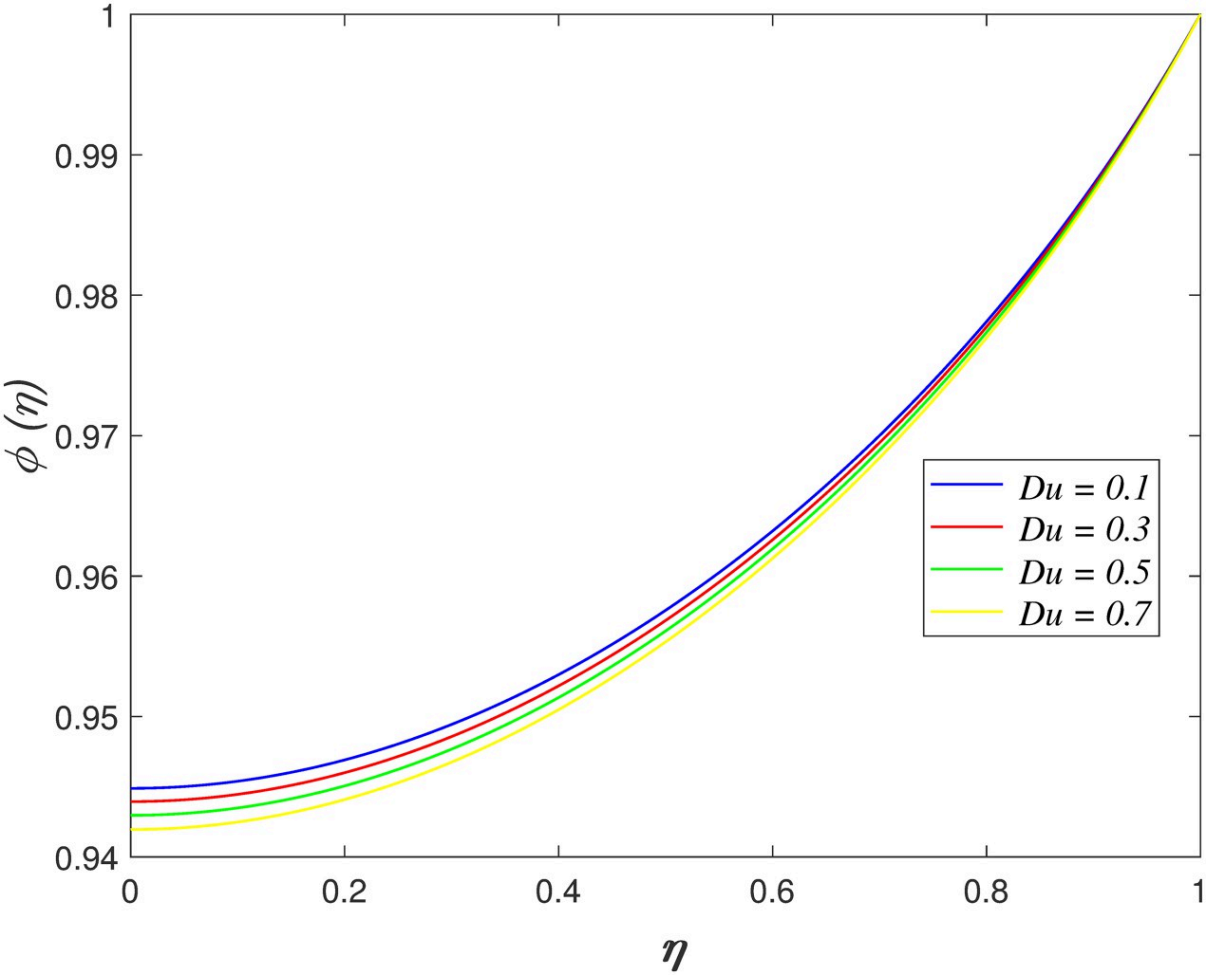
Impact of *Du* on *ϕ*(*η*).

**Fig 14:**
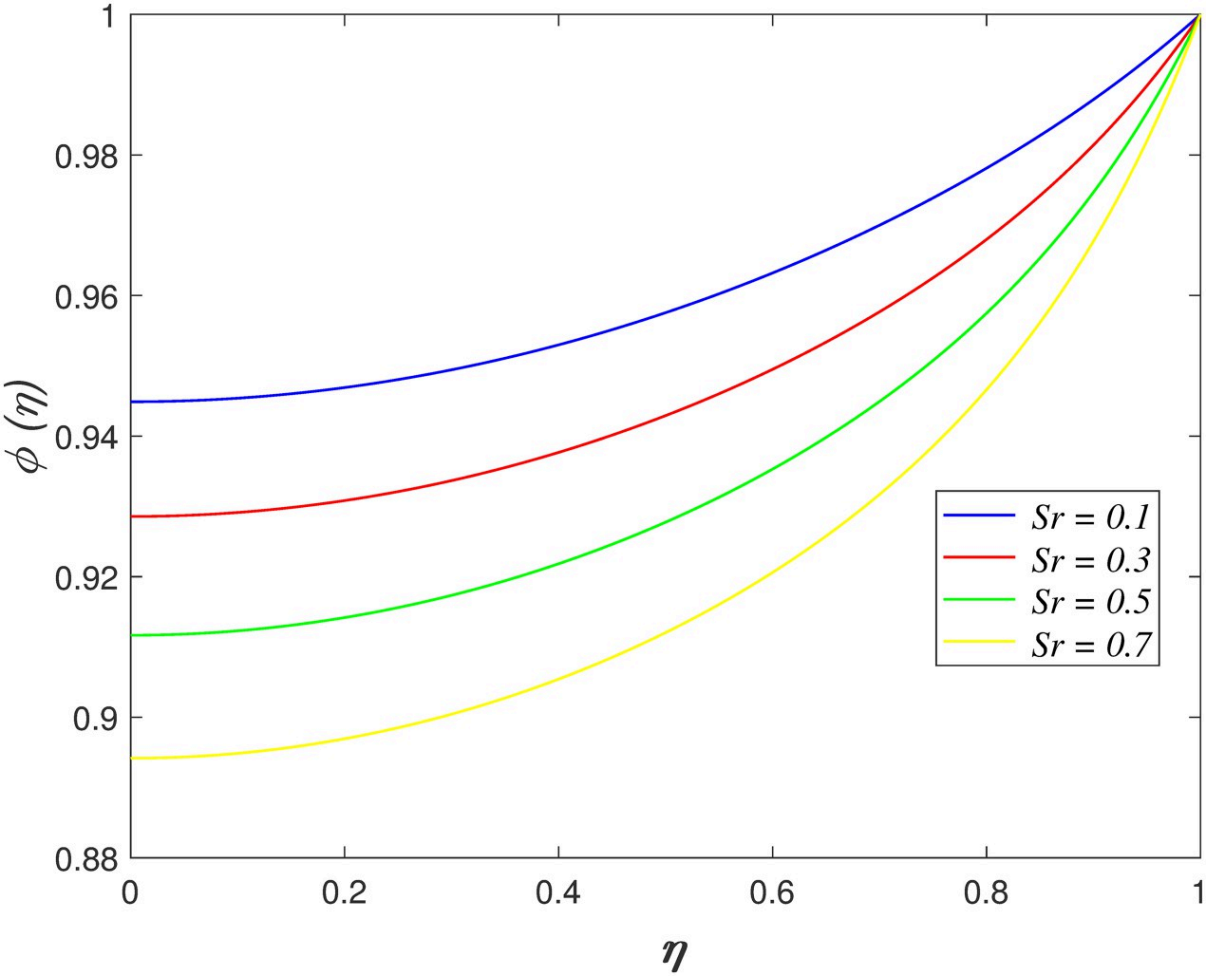
Impact of *Sr* on *ϕ*(*η*).

**Fig 15:**
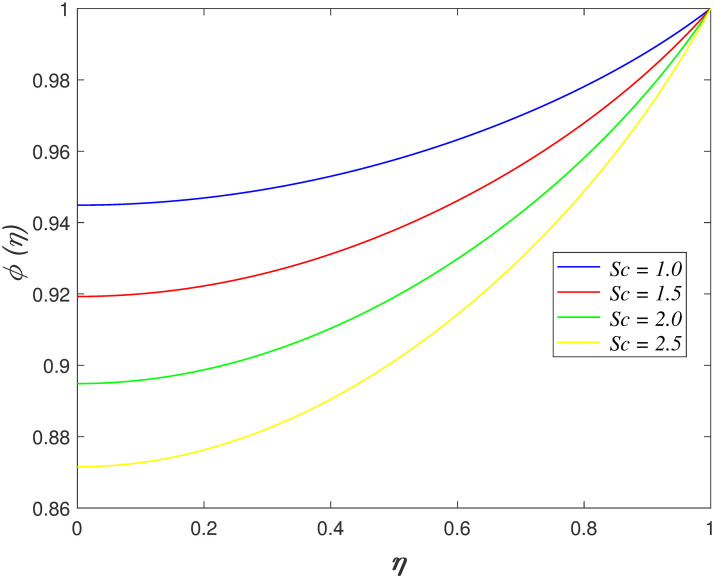
Impact of *Sc* on *ϕ*(*η*).

**Fig 16:**
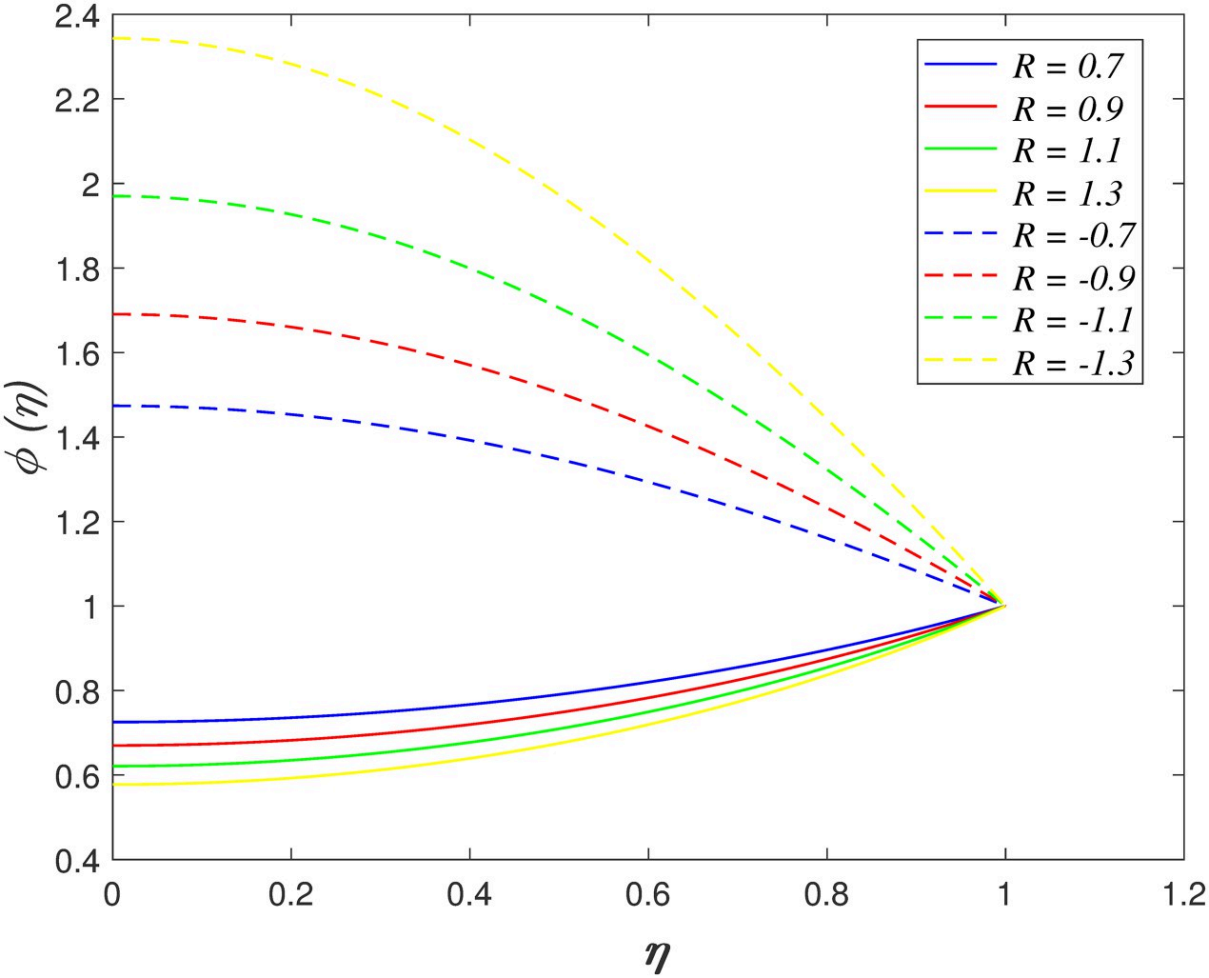
Impact of *R* on *ϕ*(*η*).

Fig 13 demonstrates the impacts of *Du* on concentration region. The concentration in the flow drops for increasing *Du*. Dufour effect is known as the energy flux generated by concentration differences. It has result in the increment of fluid temperature by lowering the concentration in the flow. The variation of *Sr* on concentration region is portrayed in Fig 14. The concentration profile declines as *Sr* rises. Soret effect or thermal diffusion is the mass flux generated by temperature differences. The mass transfer from the fluid area to the above plate accelerate due to Soret effect and thus, decreasing the fluid concentration. Fig 15 explores the variation of *Sc* on concentration region. The concentration reduces with raise in *Sc*. The mass diffusion slows down when *Sc* increases. The decrease in mass diffusivity from the surface to the fluid flow result in the concentration drops. The influences of *R* on concentration region is illustrated in Fig 16. The chemical reaction impacts are characterised by destructive (*R* > 0) and constructive (*R* < 0). It is explored that the concentration region increasing with (*R* < 0) and it decelerates with (*R* > 0). The increment of constructive chemical reaction enhances the reaction rate in the boundary layer. This phenomenon raises the fluid concentration. The same behavior of concentration profiles is observed with impacts of *Du*, *Sr*, *Sc* and *R* in the previous results by Naduvinamani and Shankar [50].

## 4 Physical quantities of fluid flow

Physically, the dimensionless parameters in the fluid are skin friction, Nusselt and Sherwood terms. The friction force at the surface boundary is described by skin friction. Moreover, Nusselt and Sherwood terms represent the rate of thermal and mass transfer in the fluid and surfaces. The terms of *Cf*_*x*_, *Nu*_*x*_ and *Sh*_*x*_ are denoted by [64]

$$\begin{array}{c} Cf_{x}=\frac{\mu_{B}(1+\frac{1}{\lambda_{1}}){\left[\frac{\partial u}{\partial y}\right]}_{y=h(t)}}{\rho_{f}v_{w}^{2}},Nu_{x}=\frac{-l\alpha_{f}{\left[\frac{\partial T}{\partial y}\right]}_{y=h(t)}}{\alpha_{f}T_{w}},Sh_{x}=\frac{-lD_{m}{\left[\frac{\partial C}{\partial y}\right]}_{y=h(t)}}{D_{m}C_{w}}, \end{array}$$ (29)

The non-dimensional form of *Cf*_*x*_, *Nu*_*x*_ and *Sh*_*x*_ are

$$\begin{array}{c} \begin{array}{cc} & \frac{l^{2}}{x^{2}}\left(1-\alpha t\right)Re_{x}Cf_{x}=(1+\frac{1}{\lambda_{1}})f^{\prime \prime }\left(1\right),\\ & \sqrt{\left(1-\alpha t\right)}Nu_{x}=-\theta^{\prime }\left(1\right),\\ & \sqrt{\left(1-\alpha t\right)}Sh_{x}=-\phi^{\prime }\left(1\right). \end{array} \end{array}$$ (30)

The numerical outputs of skin friction, Nusselt and Sherwood parameters with variation of the dimensionless parameters are shown in Tables 2 to 4. The effects of *S*, λ_1_, *De*, *Ha* and *Da* on wall shear stress is displayed in Table 2. It is found that wall shear stress boosts with increment of *S* and *Ha*, in contrast it decreases for elevating *De*, λ_1_ and *Da*. The velocity rises when the plates approaching closer, which resulting in the friction force in the boundary region elevates. Besides, the Lorentz force accelerates the flow near the boundary, and consequently enhances the frictional force in the flow. Meanwhile, the raise in viscosity of Jeffrey fluid owing to the higher values of λ_1_ and *De* strengthen the intermolecular force of fluid particles. It has caused the fluid velocity slowing down. Next, the flow over porous medium encounter opposition from the drag force near the boundary. Thus, the drop of velocity profile result in the wall shear stress declines. Table 3 presents the impacts of *Pr*, *Ec*, *R*_*d*_, *γ* and *Du* on heat transfer rate. It is noticed that *Pr*, *Ec*, *R*_*d*_, *γ* and *Du* elevate the Nusselt number. The ratio of convection heat transfer and diffusion heat transfer is described by Nusselt number. The increment of *Pr*, *Ec*, *R*_*d*_, *γ* and *Du* accelerate the kinetic energy, which cause the temperature and convective heat transfer enhances in the flow boundary. Meanwhile, the temperature reduces when *R*_*d*_ increases as shown in Fig 9. The presence of thermal radiation raises the heat transfer from the flow area to the boundary and hence, decreasing the temperature profile. This behaviour promotes the heat transfer rate on the boundary region. The variation of *Sc*, *R* and *Sr* on Sherwood number is exhibited in Table 4. The rate of mass transfer enhances for increasing *Sc*, *R* and *Sr*. The ratio of convection mass transfer to the diffusion mass transfer is denoted as Sherwood number. The drop of concentration profile in Figs 14 to 16 implies that the mass transfer by diffusion decelerates with increase in *Sc*, *Sr* and *R*. Thus, it indicates that this phenomenon boosts the Sherwood number and the convective mass transfer.

**Table 2:** Numerical results of −(1 + 1/λ_1_)*f*′′(1) for *S*, *De*, *Da*, λ_1_ and *Ha* when *Du* = *δ* = *R*_*d*_ = *Ec* = *Sr* = 0.1, *γ* = 0.01, *S* = 1 and *Pr* = *R* = *Sc* = 1.5.

*S*	λ_1_	*Ha*	*Da*	*De*	−(1 + 1/λ_1_)*f*′′(1)
-1.5	1.5	0.1	1.0	0.01	4.215636
-1.0					4.618600
-0.5					4.988290
0					5.330604
0.5					5.650016
1.0					5.949993
1.5					6.233272
1.0	1.0	0.1	1.0	0.01	7.022035
	1.5				5.949993
	2.0				5.413036
	2.5				5.090449
	3.0				4.875182
	3.5				4.721301
1.0	1.5	1.0	1.0	0.01	6.112595
		1.5			6.312200
		2.0			6.581781
		2.5			6.913097
		3.0			7.297395
		3.5			7.726136
1.0	1.5	0.1	1.0	0.01	5.949993
			1.5		5.856893
			2.0		5.809823
			2.5		5.781412
			3.0		5.762399
			3.5		5.748783
1.0	1.5	0.1	1.0	0.010	5.949993
				0.011	5.948999
				0.012	5.948016
				0.013	5.946997
				0.014	5.868722

**Table 3:** Numerical results of $-\left(1+\frac{4}{3}R_{d}\right)\theta^{\prime }\left(1\right)$ for *R*_*d*_, *Pr*, *γ*, *Ec* and *Du* when *Sr* = *Ha* = 0.1, *De* = 0.01, *Da* = *S* = *δ* = 1 and *R* = λ_1_ = *Sc* = 1.5.

*Pr*	*Ec*	*R*_*d*_	*γ*	*Du*	$-\left(1+\frac{4}{3}R_{d}\right)\theta^{\prime }\left(1\right)$
1.0	0.1	0.1	0.01	0.1	1.369991
1.5					2.013305
2.0					2.630255
2.5					3.221884
3.0					3.789211
1.5	0.1	0.1	0.01	0.1	2.013305
	0.2				3.828772
	0.3				5.644238
	0.4				7.459705
	0.5				9.275172
	0.6				11.090639
1.5	0.1	0.1	0.01	0.1	2.013305
		0.2			2.026351
		0.3			2.036991
		0.4			2.045834
		0.5			2.053298
		0.6			2.059684
1.5	0.1	0.1	-0.9	0.1	0.568809
			-0.6		0.951836
			-0.3		1.414305
			0.3		2.743691
			0.6		3.783311
			0.9		5.346708
1.5	0.1	0.1	0.01	0.1	2.013305
				0.2	2.221956
				0.3	2.435733
				0.4	2.654856
				0.5	2.879558
				0.6	3.110087

**Table 4:** Numerical outputs of *ϕ*′(1) for *Sc*, *Sr* and *R* as *De* = *γ* = 0.01, *R*_*d*_ = *Ec* = *Du* = *Ha* = 0.1, *δ* = *S* = *Da* = 1 and *Pr* = λ_1_ = 1.5.

*Sc*	*Sr*	*R*	*ϕ*′(1)
0.5	0.1	1.5	0.657871
1.0			1.106246
1.5			1.447371
2.0			1.725823
2.5			1.964194
3.0			2.175188
1.5	0.1		1.447371
	0.2		1.607869
	0.3		1.772411
	0.4		1.941177
	0.5		2.114357
	0.6		2.292157
1.5	0.1	-1.5	-10.553426
		-1.0	-2.410720
		-0.5	-0.627139
		0.5	0.755936
		1.0	1.142253
		1.5	1.447371

## 5 Conclusion

The present work examines the influences of Soret and Dufour on unsteady hydromagnetic flow of Jeffrey fluid through permeable medium with radiative heat transfer, chemical reaction and heat generation or absorption. The presence of joule dissipation and heating was studied. The flow is caused by squeeze within two surfaces. The conversion of PDEs to ODEs via similarity transformation is conducted. Keller-box technique is applied to resolve the governing equations. The impacts of *S*, λ_1_, *De*, *Ha*, *Da*, *Pr*, *R*_*d*_, *Ec*, *γ*, *Sc*, *R*, *Du* and *Sr* on velocity, temperature and nanoparticles concentration are studied. The importance findings of the discussion are deduced as:

1. The velocity accelerating when the surfaces approach nearer (*S* > 0) and it decreasing when the surfaces separate further (*S* < 0) at the above surface.
2. The wall shear stress enhances for increasing in *S* and *Ha*, in contrast it reduces when *De*, *Da* and λ_1_ increases.
3. The velocity, concentration, and temperature drop with increment of λ_1_ and *Ha*.
4. The velocity slows down near the upper boundary for enhancing *De* and *Da*.
5. The presence of *Ec*, *γ* and *Du* raise the heat transfer rate and temperature.
6. The enhancement of *R*_*d*_ elevate the rate of heat transfer and it decreasing the temperature region.
7. The rate of mass transfer rising and the concentration dropping when *Sr* enhances.
8. The concentration boosts in the constructive chemical reaction (*R* < 0) and it declines in the destructive chemical reaction (*R* > 0).
9. The rate of mass transfer decelerates when *R* < 0 and it increases when *R* > 0.

## Abbreviations

*B*: magnetic field
*C*_*w*_: concentration at upper surface
*C*: fluid concentration
*C*_*p*_: specific heat of fluid
*c*_*s*_: concentration susceptibility
*De*: Deborah parameter
*Da*: Darcy parameter
*D*_*m*_: mass diffusivity coefficient
*Du*: Dufour parameter
*Ec*: Eckert parameter
*Ha*: Hartmann parameter
*k*_*f*_: thermal conductivity of fluid
*k*_*c*_: chemical reaction rate
*k*_1_: permeability of porous medium
$k_{1}^{*}$: mean absorption coefficient
*k*_*T*_: thermal diffusion rate
*h*: distance of two surfaces
*l*: initial distance of two surfaces
*Pr*: Prandtl number
*q*_*r*_: radiative heat flux
*Q*: heat generation/absorption coefficient
*R*_*d*_: thermal radiation
*R*: chemical reaction parameter
*S*: squeeze number
*Sc*: Schmidt number
*Sr*: Soret number
*t*: time
*T*_*w*_: temperature at upper surface
*T*: fluid temperature
*v*_*w*_: velocity at upper surface
*u*: velocity (*x* direction)
*v*: velocity (*y* direction)
(*x*, *y*): cartesian coordinates
*α*_*f*_: thermal diffusivity of fluid
*α*: constant
*f*: dimensionless velocity
*δ*: dimensionless length
*η*: boundary layer thickness
*γ*: heat generation/absorption
*θ*: dimensionless temperature
*ϕ*: dimensionless concentration
*φ*: porosity of porous medium
*σ**: Stefan-Boltzmann constant
*σ*: electrical conductivity
λ_1_: ratio of relaxation to retardation times
λ_2_: retardation time
*ν*_*f*_: kinematic viscosity
*ρ*_*f*_: density of fluid
